# Pontine stroke presenting as isolated facial nerve palsy mimicking Bell's palsy: a case report

**DOI:** 10.1186/1752-1947-5-287

**Published:** 2011-07-05

**Authors:** Rishi Agarwal, Lochana Manandhar, Paramveer Saluja, Bala Grandhi

**Affiliations:** 1 Synergy Medical Education Alliance, Saginaw, MI 48603, USA

## Abstract

**Introduction:**

Isolated facial nerve palsy usually manifests as Bell's palsy. Lacunar infarct involving the lower pons is a rare cause of solitary infranuclear facial paralysis. The present unusual case is one in which the patient appeared to have Bell's palsy but turned out to have a pontine infarct.

**Case presentation:**

A 47-year-old Asian Indian man with a medical history of hypertension presented to our institution with nausea, vomiting, generalized weakness, facial droop, and slurred speech of 14 hours' duration. His physical examination revealed that he was conscious, lethargic, and had mildly slurred speech. His blood pressure was 216/142 mmHg. His neurologic examination showed that he had loss of left-sided forehead creases, inability to close his left eye, left facial muscle weakness, rightward deviation of the angle of the mouth on smiling, and loss of the left nasolabial fold. Afferent corneal reflexes were present bilaterally. MRI of the head was initially read as negative for acute stroke. Bell's palsy appeared less likely because of the acuity of his presentation, encephalopathy-like imaging, and hypertension. The MRI was re-evaluated with a neurologist's assistance, which revealed a tiny 4 mm infarct involving the left dorsal aspect of the pons. The final diagnosis was isolated facial nerve palsy due to lacunar infarct of dorsal pons and hypertensive encephalopathy.

**Conclusion:**

The facial nerve has a predominant motor component which supplies all muscles concerned with unilateral facial expression. Anatomic knowledge is crucial for clinical localization. Bell's palsy accounts for around 72% of facial palsies. Other causes such as tumors and pontine infarcts can also present as facial palsy. Isolated dorsal infarct presenting as isolated facial palsy is very rare. Our case emphasizes that isolated facial palsy should not always be attributed to Bell's palsy. It can be a presentation of a rare dorsal pontine infarct as observed in our patient.

## Introduction

Isolated facial nerve palsy usually manifests as Bell's palsy, which is commonly described as an acute peripheral facial palsy of unknown cause [1]. Bell's palsy typically presents with a sudden onset (usually over a period of hours) of unilateral facial paralysis that typically resolves over a period of six months [1,2]. Lacunar infarct involving the lower pons is a rare cause of solitary infranuclear facial paralysis [3]. The currently presented unusual case is one in which the patient appeared to have Bell's palsy but turned out have a pontine infarct.

## Case presentation

A 47-year-old Asian Indian man with a medical history of hypertension presented with nausea, vomiting, generalized weakness, facial droop, and slurred speech of 14 hours' duration. His physical examination revealed that he was conscious, lethargic, and had mildly slurred speech. His blood pressure was 216/142 mmHg. Tachycardia was present.

His neurologic exam showed that he had loss of left-sided forehead creases, inability to close his left eye, left facial muscle weakness, rightward deviation of the angle of the mouth on smiling, and loss of the left nasolabial fold. His afferent corneal reflexes were present bilaterally. All but seven cranial nerves were intact.

Computed tomography (CT) of the head without contrast enhancement and a CT angiogram of the head and neck showed no evidence of intra-cranial hemorrhage or ischemia. MRI of the head was initially read as negative for acute stroke. Bell's palsy was considered in the initial differential diagnosis but appeared less likely because of the acuity of the patient's presentation, encephalopathy-like imaging, and hypertension.

**Figure 1:**
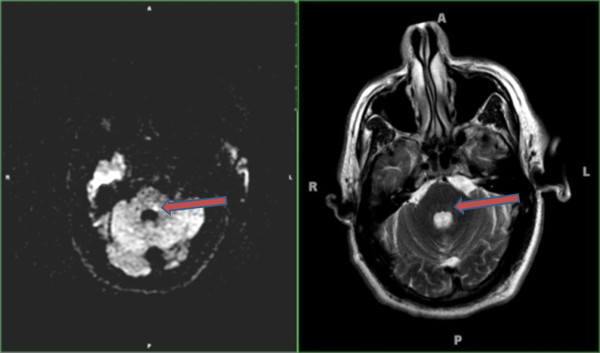
**Axial diffusion-weighted MRI and T2-weighted MRI scans**. On the day of presentation, axial diffusion-weighted MRI (left) showed a tiny focus of restricted diffusion in the left dorsal pons (arrow). Also, there was a corresponding subtle focus of signal hyperintensity noted on the T2-weighted fluid-attenuated inversion recovery sequence, which suggests a very tiny 4 mm infarct.

The MRI was reevaluated with a neurologist's assistance, which revealed a tiny 4 mm focus of restricted diffusion involving the left dorsal aspect of the pons, consistent with a tiny ischemic infarct (Figure 1).

The final diagnosis was isolated facial nerve palsy due to lacunar infarction of the dorsal pons and hypertensive encephalopathy. A transesophageal echocardiogram showed an atrial septal defect with bi-directional shunt. The patient was kept in the intensive care unit for blood pressure management and was started on aspirin, heparin, and coumadin therapy because of the remote possibility of a paradoxical embolism. There was no evidence of deep vein thrombosis in his legs.

## Discussion and conclusion

**Figure 2:**
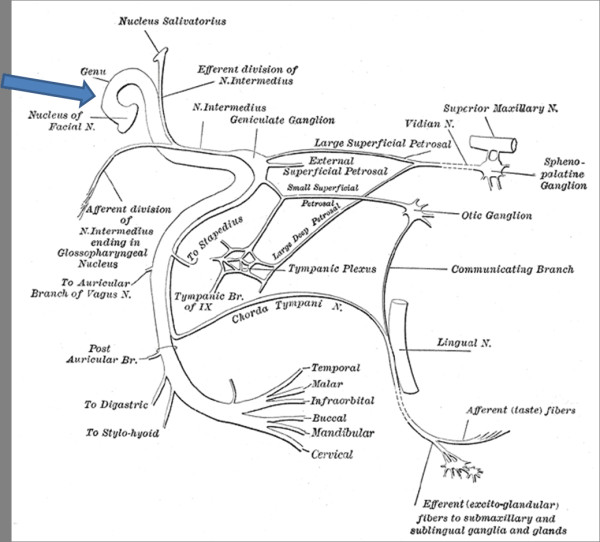
**Course of the facial nerve **[4]. Arrow indicates the site of infarct. The facial motor nucleus is located in the lower third of the pons. The nerve roots, after arising from the motor nucleus, pass around the abducens nerve nucleus as they emerge from the brainstem. In our patient, the infarct affected the part between the facial nerve motor nucleus of and the abducens nerve nucleus sparing the abducens nerve. Arrow indicates the site of the infarct.

The facial nerve is a mixed cranial nerve with a predominant motor component which supplies all muscles concerned with unilateral facial expression. Knowledge of its course is vital for anatomic localization and clinical correlation (Figure 2) [4]. Bell's palsy accounts for around 72% of facial palsies [2]. Other causes, such as tumors and pontine infarcts, can also present as facial palsy.

Pontine infarcts form around 7% of all ischemic strokes, and isolated pontine strokes contribute to around 15% of all posterior circulation infarcts [5]. Mostly, they are lacunar infarcts involving basilar artery perforators and other posterior circulation small vessels [3], with hypertension being a major risk factor [3,6].

MRI diffusion-weighted imaging (DWI) has been shown to have an advantage over CT in the detection of acute ischemic stroke, but even DWI can lead to false-negative diagnoses within the first 24 hours after presentation. Such false-negative findings are more common in strokes involving the posterior circulation, including the brainstem. In a study of 139 stroke patients, Oppenheim *et al. *[7] found that 5.8% patients had negative MRI findings within the first 24 hours after presentation. Repeat MRI performed after 24 hours of onset may be helpful in detecting such lesions which are initially negative on MRI [7].

Isolated dorsal pontine ischemia presenting as isolated facial palsy is very rare, and a review of the literature disclosed only one previously reported case [8]. Our case emphasizes that isolated facial palsy should not always be attributed to Bell's palsy. It can be a presentation of a rare dorsal pontine infarct as observed in our patient.

## Abbreviations

CT: computed tomography; DWI: diffusion-weighted imaging; FLAIR: fluid-attenuated inversion recovery; MRI: magnetic resonance imaging.

## Consent

Written informed consent was obtained from the patient for publication of this case report and any accompanying images. A copy of the written consent is available for review by the Editor-in-Chief of this journal.

## Competing interests

The authors declare that they have no competing interests.

## Authors' contributions

RA cared for the patient, did the literature search, and wrote the manuscript. LM and PS helped in the literature search and manuscript writing. BG was the attending physician and helped in the manuscript writing. All authors read and approved the final manuscript.
